# Alpha-gal syndrome and the gastrointestinal reaction: a narrative review

**DOI:** 10.3389/falgy.2025.1535103

**Published:** 2025-01-24

**Authors:** Susan B. H. Propst, Dorothea K. Thompson

**Affiliations:** ^1^ School of Pharmacy, College of Pharmacy and Health Sciences, Campbell University, Buies Creek, NC, United States; ^2^ Department of Pharmaceutical and Clinical Sciences, College of Pharmacy & Health Sciences, Campbell University, Buies Creek, NC, United States; ^3^ Department of Pharmaceutical Sciences, School of Pharmacy, South College, Knoxville, TN, United States

**Keywords:** alpha-gal syndrome, galactose-α-1,3-galactose, alpha-gal, food allergy, gastrointestinal symptoms, meat allergy, IgE

## Abstract

Gastrointestinal (GI) disturbances such as abdominal pain, nausea, and diarrhea are infrequently attributed to food allergies as an initial diagnosis in the absence of more traditional allergic reactions like hives, angioedema, or anaphylaxis. Alpha-gal syndrome (AGS) is an atypical and under-recognized allergy characterized by a delayed hypersensitivity reaction to the oligosaccharide galactose-α-1,3-galactose, a carbohydrate found in non-primate mammalian meat and derived products. This review of the current literature on AGS focuses on GI manifestations and diagnostic challenges. While clinical presentations of AGS vary widely, predominant or isolated GI symptoms, when manifested, can overlap with other disorders, thus making a timely and accurate diagnosis challenging. Here we provide an updated review of the epidemiology, pathophysiology, clinical presentation, and management of AGS. Current diagnostic approaches, treatment strategies, and areas requiring further research are also discussed.

## 1 Introduction

Alpha-gal syndrome (AGS), also known as alpha-gal allergy or red meat allergy, is an immune-mediated condition characterized by a delayed hypersensitivity reaction to galactose-α-1,3-galactose (alpha-gal), an oligosaccharide present in non-primate mammalian meat (e.g., beef, pork, or lamb) and mammalian-derived products containing alpha-gal (e.g., gelatin-rich candies, diary food products, therapeutic monoclonal antibodies, vaccines) (1, 2). The alpha-gal epitope is synthesized by the glycosylation enzyme *α*1–3galactosyltranferase (*α*1–3GT) (3). While the alpha-gal moiety is present in most mammals, the carbohydrate is absent in humans and some primates due to inactivation of the gene encoding the *α*1–3GT enzyme (3, 4). Therefore, the alpha-gal epitope is highly immunogenic in humans (5) and confers allergenicity to various molecules present in foods and pharmaceuticals (reviewed in 1, 2). Current evidence suggests that primary immune sensitization to alpha-gal occurs via tick bites, which introduces the antigenic glycan into the host's bloodstream and induces the formation of a serum specific immunoglobulin E (IgE) to alpha-gal (see 2 and (6) for recent comprehensive reviews). Subsequent consumption of red meat and/or dairy products may elicit symptoms such as anaphylaxis, urticaria, pruritus, angioedema, and gastrointestinal (GI) disturbances in sensitized individuals (7, 8). Conventional IgE-mediated food allergies are characterized by an IgE antibody response to a specific protein and immediate type I hypersensitivity reactions; however, the novel immune response to the alpha-gal oligosaccharide creates new clinical challenges. For example, AGS can take years to develop, and symptom onset can occur at any age, often leading to delays in obtaining an accurate diagnosis (9, 10). This review aims to synthesize the current understanding of AGS, including clinical presentation, diagnosis, and current approaches to management. We give special consideration to the GI phenotype of this disease and the difficulty in accurate diagnosis for those who present with symptoms focal to the GI tract.

## 2 Epidemiology

AGS was discovered in the early 2000s when patients experienced unexpected reactions after receiving the anticancer agent cetuximab, a chimeric mouse-human IgG1 monoclonal antibody (11, 12). These patients were symptomatic with alpha-gal exposure, even though they had no known red meat allergy prior to initiating cetuximab treatment. In a study of 76 case patients receiving cetuximab, 25 had a hypersensitivity reaction to the drug and some experienced severe episodes of anaphylaxis or angioedema; of those, 17 had IgE antibodies specific to galactose-α-1,3-galactose before treatment was initiated (12). The alpha-gal motif is present on the heavy chain Fab portion of the cetuximab structure (12). Around the same time, researchers in Virginia (USA) and Australia noted that patients with a history of tick bites presented with anaphylaxis after ingesting mammalian meat (13, 14). Commins et al. (7) reported that hundreds of cases involving delayed anaphylactic reactions to red meat could be linked to the presence of IgE antibodies specific to the alpha-gal oligosaccharide (7). From 2010 to 2018, more than 34,000 cases of suspected AGS were documented in the U.S (15). A recent study analyzing data collected over the observational period of 2017–2021 showed an annual increase in positive alpha-gal–specific IgE antibody tests of 15,000 each year during that time (16). Positive cases clustered predominantly in counties located in the southern, midwestern, and mid-Atlantic U.S (16).

The production of IgE antibodies to mammalian alpha-gal has been linked to ectoparasitic exposure (13, 14, 17–20). In the U.S., the bite of the Lone Star tick (*Amblyomma americanum*) is strongly associated with induction of clinical AGS (14). The geographic range of the Lone Star tick covers primarily the southeastern and midwestern area of the U.S. but is expanding northward and even into Canada (21, 22). Climate change has been implicated in the expansion of the Lone Star tick, allowing a larger endemic range, increased tick populations, and longer active periods (21). The typical geographic range for the Lone Star tick coincides with areas in the U.S. where suspected AGS cases are most prevalent (16).

Systematic epidemiological studies on AGS are limited. Individuals most at risk are those who live in the southeastern U.S. and spend a large amount of time outdoors (e.g., forest workers and hunters) (23). A case-control study found that AGS patients were 11.2 times more likely to recall a history of tick exposure, indicating tick bites as a risk factor for AGS and increased levels of alpha-gal–specific IgE (24). Other risk factors for alpha-gal sensitization include older age (aged 50 and older) and atopy (23, 25). Additionally, frequent tick exposure appears to pose a greater risk for elevated alpha-gal IgE than a single prolonged exposure during tick attachment (25).

## 3 Pathophysiology

Numerous studies conducted in the USA, Europe, Australia, Japan, and Brazil have contributed to the mounting evidence establishing a connection between bites from specific tick species and the development of AGS (13, 14, 17–20). Crispell et al. (9) employed N-glycome profiling and immunoproteomic analysis to demonstrate the presence of terminal *α*-1,3-galactose residues in the saliva and salivary glands of the Lone Star tick (*Am. americanum*) and the black-legged tick (*Ix. scapularis*), but not in the species *Amblyomma maculatum*. This study and others (26, 27) support the idea that antigens containing alpha-gal epitopes are transmitted to the host via tick saliva when the tick bites, thus creating a risk for AGS development. Recently, evidence from observational studies (24, 28), a systematic review (29), and experimental murine model studies (30, 31) suggest that tick bite exposures lead to alpha-gal–specific IgE sensitization in humans. Choudhary and coworkers (30), for instance, reported that knockout mice deficient in *α*1–3GT (AGKO mouse model), mimicking “alpha-gal-deficient” humans, generated alpha-gal–specific IgE following subcutaneous injection with *Am. americanum* tick salivary gland extract. Subsequent studies using the same alpha-gal-deficient mouse model demonstrated a significant increase in total IgE, IgG1, and alpha-gal IgG1 in AGKO mice sensitized by Lone Star tick bites compared to non-sensitized mice (31). In both studies, sensitized AGKO mice displayed a systemic allergic reaction, measured as a drop in core body temperature, after oral pork challenge (30, 31). Furthermore, researchers have observed augmented anti-alpha-gal IgE antibody levels in individuals exposed to repeated tick bites (14, 20, 32).

Despite increased research attention on AGS, the immunopathogenic mechanisms underlying alpha-gal sensitization and the effector phase leading to delayed allergic responses are not fully understood. For instance, how clinically relevant ticks induce a pro-allergic Th2 immune response to alpha-gal requires further elucidation. The availability of several animal models (30, 33, 34) for alpha-gal allergy should help to address these mechanistic gaps in the future. Two hypotheses have been proposed to explain the mechanism leading to anti-alpha-gal IgE antibody production following a tick bite (35). The first hypothesis posits that alpha-gal antigens on tick saliva proteins are presented to dendritic cells (DCs) and B cells in the “context of Th2 cell-mediated immunity” induced by exposure to tick saliva components (35). The second hypothesis suggests that factors like prostaglandin E2 (PGE_2_), which is present at abundant levels in tick saliva, and other bioactive molecules in tick saliva polarize cytokine production of DCs toward a Th2 phenotype, thus promoting isotype class switching of B cell clones from IgM/IgG to IgE production (35, 36). Previous studies support the idea that sensitization is driven by a combination of alpha-gal antigen and tick salivary factors (36, 37). Sharma and colleagues (31) observed increased expression of Th2 signature cytokine genes *Il33* and *Il4* and their respective receptors after AGKO mice were subjected to tick infestations, supporting Th2 polarization. Using *α*-gal knockout pigs, Wang et al. (34) found increased Th2 cell infiltration of the skin following sensitization. In a recent *in vitro* study, tick protein extract activated a Th2-skewed cytokine profile in peripheral blood mononuclear cells from AGS patients in contrast to healthy controls (38).

The initial encounter between tick-injected saliva antigen and host immune cells occurs at the skin epithelial barrier. Resident dendritic cells in the skin recognize, capture, and process salivary *α*-gal antigens and migrate to skin-draining lymph nodes to initiate the sensitization of B cells (39, 40). Tick antigen-specific B cells can migrate to the gut and further differentiate into IgE-secreting plasma cells (39). Mast cells, which are abundant in the GI tract, and basophils express IgE-binding receptors. When mammalian meat is ingested and absorbed, the alpha-gal epitope binds to the alpha-gal–specific IgE present on sensitized basophils and mast cells. Activated mast cells degranulate and release allergy-specific mediators like histamine and pro-inflammatory cytokines which act on smooth muscle, exocrine glands and neurons, causing pain and mucus production (41).

## 4 GI phenotype of AGS

AGS can present with a spectrum of clinical manifestations, including pruritus, urticaria, angioedema, anaphylaxis, and GI symptoms such as abdominal cramping/pain, diarrhea, nausea, and emesis. The present review focuses on the GI symptoms of AGS, as patients can often be misdiagnosed with other conditions, such as irritable bowel syndrome, or other diseases of gut-brain origin (42, 43). Symptoms commonly associated with irritable bowel syndrome (IBS), such as abdominal pain, nausea, vomiting, and diarrhea, are also associated with AGS. These symptoms can overlap with other GI disorders, such as Crohn's disease and colitis, and often make the diagnosis of AGS more challenging, especially in patients who have no self-reported recent history of tick exposure (42).

The oligosaccharide epitope alpha-gal is present in both glycolipids and glycoproteins, and a correlation has been noted between the length of time to digest glycolipids and the delayed onset of allergic symptoms in some patients, although the exact reason has not been identified (44). Some patients have been able to eat lean meat with no reaction and other times have a severe reaction eating meats with higher fat content (44). Research suggests that lipids may be the rate-limiting step in the delayed allergic response since lipids enter the bloodstream 3-6 h after ingestion (44). The conversion of lipids into chylomicrons and then into low-density lipoproteins also may play a role in delayed symptom onset (44). In some cases, the alpha-gal reaction diminishes over time; however, repeat tick exposure can reverse this decrease in sensitivity back to its original reaction severity or worse (45). Variations in clinical presentation have been observed among AGS patients (43). The delayed onset of allergic response after food consumption is unique to alpha-gal, but individual reactions are variable among sufferers of this syndrome, and this can often lead to misdiagnosis (10, 42). The reaction frequencies and alpha-gal dose required to cause clinical symptoms also vary, with some sensitized individuals remaining asymptomatic when consuming meat products and others developing a major reaction to even small amounts of alpha-gal–containing food products. Although alpha-gal can vary in amounts among different foods, higher concentrations exist in the organs of pork and beef products. If a person consumes a fattier meal, they tend to show more consistent hypersensitivity reactions, and cooking has no apparent effect on the antigenic determinant of alpha-gal (30, 46). Other factors involved in absorption and a lower threshold of response include exercise, the use of anti-inflammatory products, repeated tick bites, and alcohol consumption (47–49).

Table 1 summarizes key findings of several studies involving patients with GI symptoms who were treated at separate GI clinics. In a recent retrospective study, investigators reviewed the electronic medical record data for 1,112 adult patients who underwent alpha-gal IgE testing, of which 359 patients (32.3%) tested positive for serum IgE to alpha-gal, and 270 (24.3%) stated their main complaint was abdominal symptoms such as pain, nausea, diarrhea, and vomiting, commonly delayed 3–6 h after ingestion of mammalian products (42). Patient descriptions of abdominal pain varied from generalized epigastric to smaller incidences in the left or right quadrants, periumbilical, and suprapubic areas. When these patients followed an alpha-gal–free diet, 82% reported improvement of their GI symptoms. Also noteworthy, 19 patients in this study were previously diagnosed with IBS, and of those, 14 tested positive for alpha-gal–specific IgE antibodies. The researchers concluded that clinicians, especially those practicing in the Lone Star tick habitat, should consider AGS in cases of GI symptoms of unknown etiology, even in the absence of skin and respiratory allergy symptoms (42).

**Table 1:** Key findings in the AGS literature on GI symptoms, prevalence of alpha-gal sensitization in screening population, and predictive values.

Key aspects	Findings and sources
Gastrointestinal Symptoms	• In a study of two GI clinics, 16 patients had elevated serum alpha-gal IgE levels and GI complaints without associated rash or anaphylaxis; 75% of these patients recalled a tick bite and median time from symptom to diagnosis was reported to be 21 months (43). • In a study of 91 AGS patients who ate meat under observation in two clinical trials, 72.5% developed one or more GI symptoms, with 40.7% developing GI-isolated symptoms (50). • In a study investigating alpha-gal sensitization among allergy clinic patients with allergic history, 64% of children ages 4–17 presented with GI/oral symptoms (51). • In an observational study of 245 alpha-gal–positive patients who presented to allergy clinics due to meat allergy, 64% reported GI symptoms after ingestion of mammalian meats (52). • In a retrospective study of 40 alpha-gal–positive children in allergy clinics, 77.5% reported GI symptoms alone, with 54% of those having full or partial resolution of symptoms by diet elimination (53). • Carrageenan was linked to GI symptoms in alpha-gal, IBS, and UC patients due to the alpha-1→3 galactosidic bond that is recognized as foreign by human immune systems (54).
Prevalence of Alpha-Gal Sensitization in Screening Population	• In a cross-sectional study of forestry service employees in southwest Germany, a 35.0% alpha-gal positive prevalence was determined among 300 subjects using a ≥0.10 kU/L cutoff (23). • Serum assays from 26.3% of 118 subjects presented for cardiac catheterization showed IgE to alpha-gal (55). • U.S. screening population of 404 colonoscopy participants showed 31.4% had elevated serum alpha-gal IgE > 0.1 kU/L and reported similar incidence of abdominal pain. However, participants with levels >2 kU/L showed no difference in mammalian meat intake when compared to participants who were seronegative (56). • Of screened incoming U.S. military personnel (3,000 total of which 81.9% were male), prevalence of alpha-gal IgE was 6.0%, with the highest rates observed among recruits from Arkansas (39%), Oklahoma (35%), Missouri (29%), and several other southeastern states (>10%). Alpha-gal sensitization was associated with the male sex, rural residence, and White race (57).
Predictive Values	• In a cohort study of 84 participants diagnosed with AGS, IgE >5.5 kU/L was associated with a 95% probability of having a meat allergy (58).

In a study documenting the time course of clinical symptoms following an oral food challenge, Commins et al. (59) found that AGS reactions occurred 3–6 h after ingestion of mammalian meat. Symptoms ranged from pruritus, urticaria and abdominal cramping to cough, chills, and chest tightness. Among 91 alpha-gal–allergic patients (including both children and adults) who exhibited symptoms after oral food challenge, 71% presented with abdominal pain and 40.7% presented with GI-isolated symptoms (50). In a retrospective cohort study, the most common symptoms reported among alpha-gal IgE-positive patients from 2 GI clinics in North Carolina were abdominal pain (87.5%), nausea (75%), and diarrhea (68.75%) without accompanying skin, respiratory, or circulatory symptoms (43). Additionally, GI manifestations of AGS have been observed in pediatric populations. Among pediatric patients exhibiting delayed red-meat allergy, two studies reported a prevalence of GI symptoms of 64% and 66% in combination with other AGS reactions such as urticaria and angioedema with or without anaphylaxis (51, 52). In a retrospective investigation of 199 pediatric patients, Busing et al. (53) reported that 20.1% tested positive for alpha-gal and presented with only GI symptoms.

## 5 Diagnosis

GI symptoms are not usually considered to be a presentation of food allergy unless accompanied by other allergic responses (50, 60, 61). Patients can endure months to years of confusion regarding their diet and undergo numerous tests only to find themselves with unanswered questions about their pain. One of the authors (S.P.) of this review spent two years exploring possible causes of abdominal pain, diarrhea, and nausea without any clinical suspicion that the disease was AGS. Prior to a definitive diagnosis, the author underwent endoscopy and colonoscopy procedures, was diagnosed with IBS, and received little-to-no relief from prescribed anti-spasmodic and anti-inflammatory drugs. After an official diagnosis via a positive blood serum test for the presence of alpha-gal–specific IgE antibodies, the author was able to achieve full resolution of symptoms by adhering to an alpha-gal–free diet, including avoidance of beef, lamb, pork, dairy, magnesium stearate, and carrageenan. Carrageenan, an additive used to emulsify and stabilize food, has links to AGS due to a similar epitope (54, 62). While not structurally analogous to alpha-gal, magnesium stearate preparations may contain residual alpha-gal derived from animal sources often not known or not disclosed (1). The diagnosis of gastrointestinal alpha-gal syndrome has been proposed among people with consistent symptoms, an elevated alpha-gal IgE titer, and adequate response to GI symptoms with alpha-gal avoidance (8).

It should be noted that an exact level or range of IgE alpha-gal titer to diagnose AGS has not yet been clinically established (Table 1). However, recent clinical guidelines by the American Gastroenterological Association (AGA) recommend screening for AGS using an IgE alpha-gal test for those patients who present with recurring episodes of nausea, vomiting, abdominal pain or diarrhea, especially if the patient spends time outdoors or has had a recent tick bite. After such testing and an adequate symptom response to two months of alpha-gal avoidance, the diagnosis of alpha-gal is then supported (8).

The most rigorous way to substantiate the food allergy in AGS is to perform an oral food challenge. Traditionally, food allergy testing introduces small amounts of food allergen over time; however, the delay in symptom onset for alpha-gal patients may require a larger allergen dose to illicit a response (59). In the food challenge conducted by the University of Virginia and Duke University, investigators acknowledged that a non-double-blind, placebo-controlled study was a limitation, but this format was used as a precautionary measure to avoid giving a secondary dose after a significant delayed response in these patients. The researchers concluded that the clinical symptoms observed in patients overlap with basophil activation due to probable antigen presence in the serum (59). Recently, Mehlich et al. (63) proposed that a basophil activation test can be used to differentiate between alpha-gal syndrome and asymptomatic alpha-gal sensitization and would be helpful to clinicians in determining if a patient requires total avoidance of the allergens. This is especially important given the uncertainty in the clinical relevance of alpha-gal sensitization, the dangers associated with oral provocation tests, and the delayed onset of reaction requiring hours of patient observation (63).

## 6 Treatment and prevention

The primary treatment for AGS is the elimination of alpha-gal–containing mammalian meats from the diet (42, 43, 53, 64, 65). Additionally, some patients should avoid fats, butter, milk, and any other products made from mammals. Alpha-gal patients are safe to enjoy fish, turkey, chicken, emu, or other fowl. Gelatin products derived from a mammal should also be avoided, such as those found in marshmallows and gummies. Collagen and some processed foods also can elicit an allergic response in alpha-gal patients, so caution should be exercised. Vitamins and supplements marketed as vegan or dairy-free may have alpha-gal ingredients unknown to the distributor or manufacturer. A dietician can be consulted, as needed, for assistance in identifying alpha-gal–containing foods, vitamins, and supplements to avoid. It may be necessary to have diphenhydramine available in case a patient accidentally consumes alpha-gal–containing products. An epinephrine pen may be prescribed by the patient's doctor if anaphylactic reactions occur (59).

The prophylactic and therapeutic efficacy of immunotherapy with nanoparticles (NPs) encapsulating alpha-gal glycoprotein has been investigated most recently in an AGKO mouse model (66). Researchers found that prophylactic treatment of AGKO mice with *α*Gal NPs reduced production of splenocyte Th2 cytokines IL-4, IL-5, and IL-13 and correlated with suppressed formation of *α*Gal-specific IgE and hypersensitivity reactions (measured by reduced basophil activation and histamine release) following intragastric challenge with beef extract (66). Therapeutic administration of *α*Gal NPs to sensitized mice showed partial efficacy with reductions in Th2 cytokine production and serum specific IgE but not in basophil activation and histamine release (66).

Avoidance of tick bites is essential because additional exposure to galactose-α-1,3-galactose may increase the severity of reactions to mammalian meats and dairy products (9, 51). Symptoms may wane over time if the risk of another tick bite and alpha-gal exposure is mitigated. Although there is no guarantee of reduced IgE levels solely due to avoidance, patients can be further sensitized by subsequent tick bites (14). It is advisable to repeat testing of IgE levels and other allergies within 6–12 months after the initial diagnosis. If levels decrease and a patient feels comfortable doing so, they can reintroduce mammalian products into their diet by test bites and careful monitoring for reactions with a trusted partner. However, if pruritus, angioedema, or anaphylaxis is present during a reaction, McGill et al. (65) suggested patients should only attempt reintroduction of alpha-gal containing products under the supervision of an allergist.

Medical devices derived from bovine or pork materials, such as artificial cardiac valves and other biosynthesized prosthetics, have been documented to cause an allergic response *in vitro* in alpha-gal patients (67). Patients have experienced early failures with devices made of porcine bio-prostheses and other newly engineered devices which have received approval from the Food and Drug Administration (FDA) (67). Therefore, providers should use caution when making decisions to use mammalian-based transplantation devices. Patients also should use caution when obtaining vaccinations as some contain pork-derived gelatin which could cause an allergic response in sensitized individuals (68).

## 7 Future studies needed

Prospective studies are needed to develop a more comprehensive understanding of AGS and identify ways to predict and improve allergic symptoms. Future prospective studies would ideally address a way to correlate the degree of dietary changes in symptomatic patients with changes in allergic response to the alpha-gal allergen (43). Further research also needs to examine the potential long-term health impacts of both symptomatic and asymptomatic alpha-gal patients, especially for prolonged exposure to inflammatory cytokines in the GI tract and cardiovascular system (55, 69). Studies have shown an association between IgE sensitization to alpha-gal and an increased risk for coronary artery disease (55, 69). In-depth studies of the *α*-1,3-galactosyltransferase present in the human gut microbiome may yield an approach to modifying the level of anti-alpha-gal antibody response (70). This would not only provide a way to treat those suffering with allergic reactions to mammalian products, but also prevent rejection of animal-engineered xenotransplantation devices (70).

Additional biomarkers or other predictive markers of response should be established as part of a screening algorithm for patients with idiopathic symptoms of abdominal pain and counseling of alpha-gal patients since there is no established algorithm specific for this group (43, 53, 58). Mabelane et al. (58) investigated IgE levels in 84 patients diagnosed with alpha-gal and determined an IgE value of 2.00 kU/L was indicative of a greater than 58% probability of having a meat allergy, whereas an IgE value > 5.5 kU/L had a 95% probability. This study also revealed two different phenotypes for alpha-gal allergic patients: one where patients react with GI symptoms only, and the other with GI distress, urticaria, rash or other severe reactions. The finding of different phenotypes provides more evidence of the need to establish protocols for screening or further testing when presented with focal GI symptoms of unknown origin (58).

## 8 Conclusion

AGS represents a significant diagnostic challenge, particularly due to its unique delayed onset and varied clinical presentation, particularly for patients who present with GI symptoms. The increasing prevalence of AGS, driven by expanding tick populations, underscores the urgent need for enhanced awareness among healthcare providers and improved diagnostic protocols (71). Improved recognition and understanding of AGS will significantly enhance patient care and quality of life for those affected by this emerging syndrome. Of particular importance is the recognition of isolated GI manifestations, which can lead to prolonged periods of misdiagnosis and unnecessary interventions. Future research should focus on establishing standardized diagnostic algorithms, investigating long-term health implications, and exploring potential therapeutic interventions.
